# Using nominal group technique to advance power assisted exercise equipment for people with stroke

**DOI:** 10.1186/s40900-021-00311-z

**Published:** 2021-09-28

**Authors:** Rachel Young, Karen Sage, David Broom, Katherine Broomfield, Gavin Church, Christine Smith

**Affiliations:** 1 Advanced Wellbeing Research Centre, Sheffield Hallam University, 2 Old Hall Road, Sheffield, S9 3TU UK; 2 Faculty of Health and Education, Manchester Metropolitan University, Manchester Brooks Building, 53 Bonsall Street, Manchester, M15 6GX UK; 3 Academy of Sport and Physical Activity, Faculty of Health and Life Sciences, Coventry University, Sheffield, UK; 4 4National Institute for Health Research (NIHR)/Health Education England (HEE) Clinical Doctoral Research Fellow, Gloucestershire Health and Care Foundation Trust and Manchester Metropolitan University, Sheffield, UK; 5 NIHR Clinical Pre Doctoral Academic Fellow, Community Stroke Service, Sheffield Teaching Hospitals NHS Trust, Beech Hill, Norfolk Park Road, Sheffield, S2 3QE UK; 6 Department of Allied Health Professions, Sheffield Hallam University, Collegiate Crescent Campus, Sheffield, S10 2BP UK

**Keywords:** Power assisted exercise, Nominal group technique, People with stroke, Digitalisation, Co-design

## Abstract

**Background:**

Power assisted exercise is accessible and acceptable for people with stroke. The potential for technological advancement of the equipment to improve the user experience has been identified. Involvement of end users and service providers in the design of health technologies is essential in determining how said technology is perceived and adopted. This project invited people with stroke and service providers to influence design features and determine machine selection in the preliminary stages of a codesign research programme.

**Aims:**

To capture the perspectives of people with stroke and professionals working with people with stroke about proposed digitalisation of power assisted exercise equipment and select machines for prototype development.

**Methods:**

Nominal group technique was used to capture the perspectives, ideas, preferences and priorities of three stakeholder groups: people with stroke (n = 3, mean age 66 years), rehabilitation professionals (n = 3) and exercise scientists (n = 3). Two questions underpinned the structure of the events; ‘What does an assistive exercise machine need to do to allow the person with stroke to engage in exercise?’ and **‘**Which machines would you prioritise for use with People with Stroke?’ Attendees were invited to cast votes to indicate their preferred machines.

**Findings:**

Synthesis of the data from the NGT identified four domains; software and interface, exercise programme, machine and accessories, setting and service. Three preferred machines from a range of nine were identified through vote counting.

**Conclusion:**

Nominal group technique directed the selection of machines to be included in the development of the proposed technology. The vision shared by users during the structured discussion shaped the subsequent steps in the design and testing of the new technology.

**Patient and service provider contribution:**

The opinions and preferences of people with stroke, rehabilitation professionals and exercise scientists were central to key decisions which will shape the digitalisation of power assisted equipment, influence future research and guide implementation of the new technologies.

## Plain English Summary

Seated power assisted exercise machines assist different combinations of movement and can help people with stroke to take part in exercise programmes. Nine seated machines are manufactured in the UK. It was identified that the machines could be improved through development of technology to detect and display the user’s physical effort during exercise. The research team successfully applied for funding to design and develop a new programme to display and measure user effort whilst exercising on the equipment. At the outset of the project the research team needed to make decisions about the capabilities of the new technology and select three machines from the range of nine to be prototyped. We used a method called nominal group technique during which end users with stroke, rehabilitation and exercise professionals were invited to structured meetings to share their ideas. At the end of the meetings the groups voted on their preferred machines. The ideas expressed during the meetings were listed and guided the ongoing development of the technology. The importance of a user-friendly interface was emphasised. The three machines which were allocated the most votes across the user groups were selected to be redesigned and developed with the new technology. The involvement of users at the outset of the design project ensured that they directly influenced the selection of machines and features of the new technology. Nominal group technique was an effective way of ensuring that all attendees had the opportunity to share their ideas and perspectives.

## Introduction

Involvement of end users and service providers in the design of health technologies is essential in determining how said technology is perceived and adopted [1]. User involvement is fundamental for creating a positive technology-based experience that is meaningful for the end-user [2]. The adoption of user involvement principles in the development of new assistive technologies generates a partnership between consumers and providers and shifts the traditional dynamic of “doing for” the user to “doing with” [3]. An understanding of the experiences and priorities of future users guides co-design projects and mixed methods are used to capture a rounded user perspective [1].

User involvement in technology design refers to an iterative, cyclic approach to product development during which clinicians, patients and carers influence and evaluate the new technology [2, 4]. The four stage Medical Device Technology framework (MDT) proposed by Shah et al. [2] emphasised continuous user involvement, starting with idea generation to visualise the new product. Patient and Public Involvement (PPI) can facilitate early collaboration between patients, service providers and researchers [5]. Effective PPI enhances transparency in research processes and is particularly crucial in the development and implementation of assistive technologies [6].

Inclusion of end users is evolving as they have become active partners in their own disease management and, as such, take part in research ‘with’ the research team rather than being participants of research done ‘to’ or ‘about’ them [7, 8]. However, reported barriers to effective and inclusive patient involvement in health technology decision making include under-representation, limited transparency and inaccessible consultation methods [9]. The reporting of PPI has been inconsistent with little information about the context, process or impact of public/end user involvement [10]. Volunteers who engage in user consultation have indicated that they would like their involvement to be more visible including during dissemination [11]. Guidelines to support the reporting of PPI have been developed to enhance awareness of PPI activities and the subsequent influence on research design, interpretation of outcomes and dissemination [10].

The boundaries between PPI and research can be blurred and different perspectives regarding the remit and reporting of PPI have been articulated [6, 10]. Qualitative research requires data collection and analysis for the purpose of an improved in-depth understanding of a topic and development of new theoretical perspectives; whereas PPI entails user group consultation to ensure end users’ active involvement in decisions about research priorities, design and conduct [12]. Integrated partnership approaches which involve data collection and analysis for the purpose of decision making and change has been identified as a third division positioned between PPI and qualitative research [12].

The MDT framework [2] was selected to underpin this iterative design project focussed on power assisted exercise (PAE) for People with Stroke (PwS). The model developed by Shah et al. [2] ensured that an inclusive approach to user involvement was sustained and disseminated from the point of preliminary idea generation to the development and testing of the new technology. The four-stage framework comprises (1) idea generation, (2) device design, (3) prototype testing, (4) field testing. This report describes the idea generation stage which was succeeded by co-design through storyboarding (stage two) and usability testing (stage three). Shah et al. [2] emphasised the importance of diversity in the identification of stakeholders and their model stipulates inclusion of professional users and expert end users. Strategies to enable PwS to engage in PPI include support and reimbursement for travel, tiered levels of involvement and dedicated professional support to manage challenges associated with communication and concentration [13].

### Power assisted exercise for people with stroke

People with limited mobility following stroke experience difficulty in accessing conventional exercise interventions and are under-represented in the growing body of evidence informing exercise for PwS [14]. PAE machines are accessible and acceptable for people with complex neurological impairment and limited mobility following stroke; the multi-directional, global movement patterns facilitated by the equipment are associated with improved mobility and user independence [15]. Users need to have independent sitting balance and be able to process brief instructions or demonstration to use the equipment safely [15].

Guidance on exercise interventions for PwS stipulate specific parameters for aerobic conditioning and resistance training to optimise physiological response [16]. PAE machines offer varied speed settings but the duration and intensity of exercise is not based upon the exercise prescription detailed in published guidelines [16]. Furthermore, the existing software does not measure or quantify the physical effort generated by the user. Real-time feedback during exercise has been associated with physical improvements amongst PwS [17]. Digitalisation of rehabilitation interventions creates the opportunity to optimise performance feedback and the potential to introduce cloud-based rehabilitation and monitoring [18]. Advancement of PAE to align the training stimulus with published guidelines for individual users and generate digitised biofeedback on user effort is currently being explored through a proof of concept co-design project. Resource planning at the outset of the project required the research team to select three priority machines from a range of nine to establish the proof of feasibility of the proposed technology. This paper reports on a user involvement exercise which aimed to capture the perspectives of service providers and PwS and rank the nine machines according to user preference during the planning phase of the project.

### Nominal group technique

Nominal group technique (NGT) is a consensus method which incorporates idea generation and collection, ordered group discussion and anonymous ranking of preference through voucher allocation by individual members [11]. NGT is a formal consensus development method based on structured group discussion; the method prevents individual participants from controlling the discussion and ensures all groups members have the opportunity to share their suggestions and opinions [19]. It aims to achieve agreement or convergence of opinion around a particular topic and gauge the strength of that convergence and may be applied to determine priorities [20].

NGT is well suited to user involvement as it promotes equal representation from all participants through chaired discussion and vote counting [11]. The translation of NGT findings into the preliminary phases of co-design projects ensures meaningful representation of the user perspective [19]. NGT has been applied previously to obtain views and gain consensus on interventions for supporting PwS and is an accessible method for participants with communication impairment which is experienced by one third of the stroke population [21–23]. NGT is particularly useful where participants are likely to have diverse views on a subject or where limited research evidence is available [20].

## Aims

The aims of the NGT process were to capture the user perspective of the proposed digitalisation of PAE equipment for PwS, identify and categorise priority design features and select three specific machines for prototype development.

## Methods

### PPI framework

Tritter [24] created a framework for conceptualising PPI which identified direct or indirect; individual or collective; and reactive or proactive models of involvement. The model of PPI adopted through this application of NGT was collective, direct and proactive as it captured a group perspective to shape and influence the focus of the proposed project. PwS, exercise scientists, rehabilitation experts and researchers came together to determine machine selection and priority design features for the advanced equipment. An ethos of collaboration was promoted through the chaired and structured discussion integral to NGT and the use of anonymous votes to determine machine selection. Separate sessions were scheduled for the professional user (PU) group and expert user (EU) group to ensure an honest exchange of opinion between the PU group and optimise empowerment amongst the EU group [2, 13].

### NGT members

At the outset of the project, a team of experts was identified to ensure representation from PwS, health care services and academia. To be considered for invitation to the PU meeting, expertise in either stroke rehabilitation, clinical exercise prescription or PAE equipment was required. Seven experts employed by the host institution comprising four physiotherapists and three exercise scientists were invited to the PU meeting**.** To be considered for invitation to the EU meeting, service users were required to have a diagnosis of stroke and experience of using PAE equipment. Five members of the service user group at the host institution who met this criterion were identified and invited to the EU meeting. The NGT meetings were chaired by the first author (RY) who is a research physiotherapist and directly supported by a Professor of Rehabilitation (KS). In addition, a specialist neurological physiotherapist (HL) with experience of qualitative research methods for PwS supported the EU meeting to facilitate communication and inclusion of all attendees [13].

### Ethical considerations

An ethically conscious approach was adopted throughout the NGT sessions held with the PU and EU groups [5]. Ethics committee approval was not required as the remit of this activity was PPI to facilitate user involvement at the outset of the programme. Written information about the meeting was circulated to potential attendees two weeks in advance. Signed permission was obtained from all attending NGT members which included authorisation to audio record the event and publish findings. The users involved in the NGT meetings were made aware that they could withdraw their contribution during or up to one week following the meeting to allow time for reflection upon the session and their contribution.

### Meeting format

The NGT meetings took place in accessible seminar rooms at the host institution. Transport was provided for attendees of the EU event and travel costs were reimbursed for attendees of the PU event [13]. Ground rules and objectives were communicated at the outset to set the tone of the session and promote a shared understanding of its context. The meeting addressed two questions and followed a structured format which comprised individual ideas generation in silence (written down), sharing one by one from each participant until no further ideas remained unsaid, structured discussion as a group to allow ideas to group into themes and individual voting (Table 1). The technique prevents individual participants from controlling the discussion and ensures all groups members have the opportunity to share their suggestions and opinions [19]. The events were recorded on an Olympus WS-811 Dictaphone and field notes were retained for analysis.

**Table 1:** Nominal group steps common across both meetings

Step	Activity
One	Introductions, NGT objectives and ground rules. Summary of the nine machines presented by lead author through PowerPoint software
Two	**Question one:** ‘What does an assistive exercise machine need to do to allow the person with stroke to engage in exercise?’
Three	Attendees write their list of ideas in silence
Four	Attendees invited in turn to share aloud their ideas, taking it in turns to offer one idea at a time from their written list until all the ideas were shared. Field notes documented on flipchart by RY
Five	Group discussion and merging of topics
Six	**Question two**: ‘Which machines would you prioritise for use with PwS?’
Seven	Attendees consider their options in silence
Eight	Attendees provided with 10 vouchers each. They were asked to allocate the vouchers across any number of machines and can indicate strength of preference through number of vouchers allocated. The machines were represented on A4 photographs laid out on the table

### Analysis

As this was a PPI activity rather than primary research, the intention was to summarise and categorise the ideas shared in response to the questions posed rather than to generate new theories or perspectives. The audio recordings were transcribed verbatim and the written lists generated by participants were copied into the transcription. The first author (RY) familiarised herself with the content through repeated reading and note taking.

Key stages which underpin qualitative content analysis were adopted to interpret and organise the information captured during the NGT events [25, 26]. Two rehabilitation experts from health care services (KB, GC) were identified to support the lead author (RY) in the organisation and categorisation of the transcripts. The three analysts independently coded and categorised the content of the transcripts. The interpretation and categorisation of the transcripts remained close to the manifest content to ensure a concrete rather than abstracted interpretation of the written and verbal suggestions [26]. Alignment of content back to the topics which had emerged during step five of the events ensured that the interpretation and categorisation of the transcripts remained close to the priorities emphasised by the NGT event attendees. Discussion between the analysts led to agreement upon four domains [27]. The content of the domains was tabulated with columns to represent the perspectives articulated by the PU and EU groups respectively.

The number of votes allocated to each machine were counted and recorded by RY at the end of each event. The count from the EU group (n = 3) was adjusted through multiplication to equalise with the proportion of votes from the PU (n = 6) group. The top three preferred machines were determined by the highest total count.

## Results

### Membership of the professional user meeting

Six out of seven invited experts in rehabilitation or exercise science attended the PU meeting. The PU group included a clinical physiotherapist, research physiotherapist, a higher degree physiotherapy graduate with a specialist interest in PAE and three exercise scientists with expertise in physiology, muscular performance and clinical populations (Table 2). The Managing Director of the PAE equipment manufacturer attended the meeting to answer any queries, observe the discussion and capture insight into the perspectives of the invited experts.

**Table 2:** PU attendees

Attendee I.D	Profession	Longevity of experience (years)	Gender identity	Specialist interest	Highest qualification
P1	Physiotherapist	19	Female	Neurological rehabilitation and technology	MSc
P2	Physiotherapist	3	Female	Rehabilitation and PAE	MSc
P3	Physiotherapist	29	Female	Neurological rehabilitation	MSc
ES1	Exercise scientist	18	Male	Physical activity for special populations	PhD
ES2	Exercise scientist	10	Male	Clinical exercise physiology	PhD
ES3	Exercise scientist	11	Male	Neuromuscular physiology	PhD

### Membership of the expert user meeting

Three out of five invited service users with a diagnosis of stroke and experience of using PAE machines attended the EU meeting. The three attendees of the EU meeting were female with one-sided weakness following stroke. Detail regarding experience of PAE is detailed in Table 3.

**Table 3:** EU attendees

Attendee number	Age	Time since stroke (years)	Gender identity	Impairment	Functional ambulation category	Experience of PAE
E1	76	12	Female	Left hemiparesis	2/5	1 session per week for previous 6 months
E2	67	5	Female	Right hemiparesis and aphasia	3/5	2 sessions per week for previous 6 months
E3	56	4	Female	Left hemiparesis	4/5	2 sessions per week for previous 6 months

*PAE* power assisted exercise

### Group discussion

Four content domains were identified through analysis of the written notes and transcripts captured during steps tree, four and five of the NGT meetings. These were; (1) software and interface, (2) exercise programme, (3) machine and accessories, and (4) setting and service. The features suggested by the expert groups associated with each of these categories are detailed in Table 4. Priorities identified across both groups are indicated in bold font.

**Table 4:** Features for PAE suggested by the expert groups (bold font indicates PU and EU priority)

	Category	Subcategory	PU priority	EU priority
Four domaims	Software and interface	Interface	**User friendly** **Clear visual display** Fun and motivating Gamification Demonstration video	**User friendly** **Clear visual display** Easy to reach
		Feedback	**Inter-session comparison** **Meaningful** **Individualised** Watts and power generated Heart rate monitoring Symmetry of effort Baseline comparison	**Inter-session comparison** **Meaningful** **Individualised** Sensitive to small effort Accurate and continuous Generate digital record Calculated calorie expenditure
	Exercise programme	Movement	**Functional and efficient** **Simple patterns** **Goal orientated** **Adjustable speed** **Reciprocal movement** Machine initiated Optimal limb alignment Resisted movement option	**Functional and efficient** **Simple patterns** **Goal orientated** **Adjustable speed** **Reciprocal movement** Multiple movements
		Physiological demand	**Improve motor control** **Soft tissue stretch** **Decrease hypertonicity** Physiological overload Cross education Eccentric and concentric Aerobic demand Progressive trajectory	**Improve motor control** **Soft tissue stretch** **Decrease hypertonicity** Manageable duration
	Machine and accessories	Accessibility	**Safe transfer on and off** Hemiplegia friendly Fits with transfer aid equipment	**Safe transfer on and off** Reachable components Able to access independently Height adjustable
		Accessories	**Bespoke limb support** **Quick release components**	**Bespoke limb support** **Quick release components** Secure walking aid storage Reach bar to secure balance
	Setting and service	Team	**Good knowledge of equipment** Ability to educate users Ability to support goal setting Ability to manage expectations	**Good knowledge of equipment** Available to help Understand movement patterns Understand limited mobility
		Environment	**Client centred service** Social and peer support	**Client centred service** Integrated therapy service

*PU* professional user

*EU* expert user

## Software and interface

This domain summarised the content relevant to the suggested features, functionality and aesthetics of the user interface to enable the user to engage in programme selection and receive feedback on their exercise performance. There were two subcategories; (a) interface and (b) measurement of performance.

### Interface

A user-friendly visual platform for the software was prioritised by both expert groups and the EU attendees highlighted the need to be able to reach the interface from either the right or left side. The PU group emphasised the importance of a fun and motivating interface using twenty-first century technology, and potential for gamification of exercise through the interface.

### Measurement of performance

Comparison of performance between sessions was identified as a priority feature by both groups. Amongst the PU group, the exercise scientists emphasised the importance of inter-session comparison and identified several options for units of measurement including watts, power, range of movement, heart rate and calorie expenditure. However, it was acknowledged that values such as watts and power may not be meaningful to end-users. The EU group also identified the potential value of inter-session comparison;

If there was something that kept…nowadays you would expect something computerised, technology. You’d log in …. it would have kept the data for you. Week by week. (E2).

The physiotherapists in the PU group specifically emphasised the potential value of feedback on symmetry in terms of the effort detected from the right and left side.

## Exercise programme

This domain encompassed the exercise stimulus created by the machine and the exercise programme relayed through the interface. There were two subcategories; (a) movement stimulus and (b) physiological demand.

### Movement stimulus

Functional, simple and efficient patterns of assisted movement were prioritised by both expert groups. The physiotherapists in the PU group and EU members highlighted the importance of machine-initiated movement for people with neurological impairment and suggested that the perfect machine would facilitate good alignment and direction of movement. The physiotherapists in the PU group and EU attendees identified reduced muscle tightness and tone as desired response to the exercise programme.

The machines need to be encouraging me to focus on extension because obviously flexion is like, well I do it far more than I want to…. I would like to see that reflected in the way that the machines work, and record the effort so you could actually do more of that which I want to do, not this (indicates flexion) because I do that plenty. (E3).

The exercise scientists suggested that the option to progress from assisted to resisted movement would be an additional asset to align with overload principle of training whereby the physical challenge is incremented to promote improved strength and fitness. The PU group identified those machines which assisted trunk movement as important as they enabled a stretch which would not be possible to achieve independently.

Well that one (side bend stepper) did so much, I was so thrilled with it that I said could she put it on for a couple minutes more? Because, from my physio, I know that my back needs stretching. Because otherwise the muscles just tighten up. (E1).

### Physiological demand

The attendees of the PU group emphasised the importance of creating physiological overload through progressive levels of physical challenge to stimulate adaptation to the demand of the exercise. Improvements in various aspects of muscular performance were specifically highlighted by the exercise scientists, alongside the option to adapt the target intensity according to fluctuations in user wellbeing;

“There needs to be options for progression and regression,” (ES2)… “Yes, so not because you can’t be bothered today but if they’ve got other issues and comorbidities and today is a bad day but they still want to be able to exercise.” (P1).

## Machine and accessories

This domain was emphasised more by the EU group; although the PU group also highlighted the importance of safe and accessible machines. This domain is subdivided into two categories; (a) accessibility and (b) features and accessories.

### Accessibility

The importance of being able to safely transition on and off the machines with minimal assistance was emphasised by both groups. The physiotherapists in the PU group specifically suggested equal access from either side and safety features to minimise risk of injury. The EU group wanted the machines to be height adjustable, to enable easier mount and dismount from the seated equipment.

I’m only five foot two inch, I need a step to get on some of the machines, getting on can feel like a workout in itself. (E3).

### Features and accessories

Bespoke support structures for the limbs, hands and feet were suggested by both groups alongside the importance of user-friendly, removable attachments for the limbs. The physiotherapists from the PU group emphasised the need for effective support of posture and alignment. Additional features suggested by the EU group included secure walking aid storage and a reach bar to enable users to secure their balance whilst mounting and dismounting the equipment.

## Setting and service

Attendees of both groups indicated the importance of types of setting and service where PAE for PwS may be offered and another category emerged as a result: (a) team and (b) environment.

### Team

Both groups identified the importance of a skilled team to support users during their programme of PAE. Essential skills of a service provider team identified by the EU group included knowledge of the machines, movement patterns and the availability of staff or volunteers to provide assistance when required. The PU group identified the ability to educate, provide reassurance and support realistic goals.

### Environment

A client centred service which was adapted to the needs of each individual was highlighted as important by both groups.

Well, sometimes, the helpers come to help me, and I’ve finished on one machine and they want to go straight onto the next…and I say I’m sorry, I’ve got to rest for a minute. Because I can’t rush from one thing to another. I need the time to get around. And then I’m aware that someone else is obviously waiting for your machine. (E1).

The EU group also identified the value of the physiotherapy led guidance when using PAE machines. The PU group emphasised the importance of social and peer support in exercise venues and the creation of an atmosphere which facilitates the development of friendships between users.

### Machine preferences

The anonymised machine preferences indicated by vouchers placed on a photograph of each machine were recorded and the votes from the EU (n = 3) group were multiplied twofold to ensure equal representation of machine preference with the PU group (n = 6). The three most popular machines were the Cross Cycle (23), Chest and Legs (22) and the Rotatory Torso (18). The least popular machines were the Tummy Crunch (4) and Seated Abductor (4).

The Side Bend Stepper was most popular amongst the EU group, gaining 7 out of the total 30 tokens available. However, only 3 tokens were placed on this machine from the PU group making it the fourth ranked machine overall. All nine machines gained a minimum of two tokens from the EU group whereas the Tummy Crunch and Seated Abductor gained no tokens from the PU group. The machines which predominantly assist limb movement gained the majority of votes from the PU group in contrast to the user by experience group who indicated more preference for those machines which assist movement of the trunk. The ranking of machines is displayed in Table 5.

**Table 5:** Voucher allocation per machine

Machine and action	Image	Token allocation PU group	Token allocation (× 2) EU group	Ranking
**Cross cycle** Pedalling motion of legs and combined flexion extension of the arms		17	3 (6)	1
**Chest and legs** Bimanual flexion and extension of legs and arms		14	4 (8)	2
**Rotatory torso** Rotation of the trunk with shoulders and pelvis moving in opposite directions		8	5 (10)	3
**Seated climber** Stepping action of legs and alternate reach upwards with arms		11	2 (4)	4
**Side bend stepper** Lateral flexion of the trunk and stepping action with legs		3	7 (14)	5
**Ab pullover** Elevation of upper limbs, flexion of trunk and hips		5	2 (4)	6
**Tricep dip leg curl** Extension of elbows and flexion of the knees		2	3 (6)	7
**Tummy crunch** Flexion of the trunk and hips		0	2 (4)	8
**Seated abductor** Horizontal abduction and adduction of shoulders and hips		0	2 (4)	8

## Discussion

The use of NGT methods generated novel ideas linked to the question and provided a focus for understanding user priorities at the outset of the co-design project. The NGT method elicited a list of suggested features to enhance the equipment and structured group discussion captured the importance of an individualised, science informed exercise programme. Expert consensus enabled the selection of three specific machines through token placement and ranking. The value of a motivating and progressive PAE programme which can be used independently was articulated by both expert groups. Areas of convergence between the expert groups were identified.

### Value of diverse user involvement

The features associated with the proposed PAE programme combine elements of exercise training and therapeutic rehabilitation. The exercise scientists emphasised the training overload principle to elicit a physiological response; whereas the physiotherapists and EU group discussed the value of efficient, goal oriented, reciprocal movement patterns. The range of priorities expressed illustrated the value of consultation with diverse experts in the preliminary phase of the co-design project. Published exercise guidelines for PwS have focussed on improvements in aerobic capacity and muscular performance [16]; whereas physical rehabilitation for PwS has prioritised quality of movement and functional recovery [28]. Assisted exercise enhances motor recovery and improves aerobic capacity for PwS [29] and therefore may bridge the historical gap which has existed between exercise and rehabilitation perspectives.

### Embracing a digitalised future

The introduction of assistive technologies in rehabilitation has the potential to enable users to gain empowerment over their recovery and move towards a therapist-directed rather than therapist-dependent rehabilitation programme. Both expert groups emphasised the importance of a user-friendly interface which can be operated independently by the end user. Prioritised suggestions included motivational features, graded progression and real time feedback. Previous exploration of home-based technology through co-design with PwS also highlighted the value of user feedback, motivational gamification and independent use [1].

Assistive technologies may be perceived as detached and impersonal [30], the importance of an individualised user experience was articulated during the group discussions. In the context of PAE, an individualised user experience would facilitate the user to develop a bespoke relationship with the interface to enable an adaptable, goal-oriented exercise experience and access personalised digital information regarding progress and achievements. The emotional response triggered by interaction with technology may be influenced by the product’s quality, function and individual meaning [1]. Customizable health technologies matched to the users’ needs and preferences has been identified as a priority [30].

Both groups embraced the prospect of a digitalised programme able to generate a numerical record of performance and achievement. The digitalisation of user data can enable remote supervision of rehabilitation programmes by healthcare professionals [1]. However, a recent study to introduce wearable technologies to monitor activity amongst exercise referral scheme participants was abandoned due to poor recruitment and retention [31]. Achieving the required balance between support, supervision and monitoring amongst older adults has been identified as a challenge by physiotherapists; individualised behaviour change interventions integrated with exercise prescription may enhance retention and reported experience [32].

### Service and setting

Discussion regarding the setting and service was initiated by attendees during both NGT events. Attendees of the expert groups envisaged a client centred, accessible facility operated by knowledgeable staff. PwS have reported perceived improvement in physical performance and participation associated with venue-based exercise [33], although leisure service providers have reported feeling under-confident with the stroke population due to challenges associated with their ability to access standard exercise equipment [34]. Assisted exercise programmes have been reported as acceptable and feasible for PwS in leisure settings and, for some participants, represented a stepping stone towards the use of conventional equipment [35].

### Preferences

The preferences shared by the members of the NGT enabled the selection of three machines from a range of nine to be prioritised for technological advancement. The EU group were all regular users of the range of equipment and their preference pattern suggested a level of appreciation for all nine machines in comparison to the PU group who indicated a greater preference for the machines which predominantly assisted limb movement. Our findings indicated that the EU group valued those machines which assist trunk movement and this highlighted the importance of capturing the perspective of the end user in decision making [12]. Trunk control is an important predictor of mobility following stroke [36] and assistive interventions have been previously recommended to address deficits in trunk movement [37]. The PU group expressed a preference for those machines which more closely resemble conventional gym equipment. The three prioritised machines include a combination of conventional and novel models, representing the views of all stakeholders. This divergence in opinion emphasised the importance of user involvement; continued engagement with PU and EU representatives will be sustained throughout the programme of research.

### Application of nominal group technique to Patient Public Involvement

This user involvement report has shown the importance of early stakeholder engagement at the outset of a programme of research aimed at improving PAE for PwS. Historically, the way in which PPI has been conducted has been criticised as being tokenistic and professional led [9]. NGT has been previously implemented in PPI activities [11] and, in our experience, enabled all attendees to share their perspectives, ideas, experiences and preferences. NGT enabled the adoption of a collective, direct and proactive model of PPI [24], which empowered PwS and service providers to shape key priorities and direct important decisions.

The research team acknowledge limitations associated with the NGT exercise reported in this paper. On reflection, a collective event which combined EU and PU representation may have generated cross fertilisation of perspectives and greater depth of discussion. Subsequent user involvement methods employed during stage two of the design project did integrate PU with EU groups. Some experience of using PAE equipment was considered necessary to be eligible as an expert by experience. This limited the number of PwS able to make an informed contribution to the events, and of the five identified experts by experience, only three were able to attend. All of the EU attendees with stroke were female and had committed to investment in their recovery through third sector exercise and rehabilitation services, the opinions and preferences expressed may not have been representative of the wider stroke population. Narrow user representation has been previously reported as a limitation in patient involvement in health technology funding decisions [9]. It was also noted that there was a gender split across the PU group, with an all-male representation from the exercise scientists and all-female group of physiotherapists. Future activities should aim for an optimal balance of representation to promote equality and diversity of perspectives shared.

However, this report exemplifies how NGT can generate a vision for an intelligent, individualised, goal orientated technology centred around users’ preferences and priorities. In the context of this project is facilitated the development of more targeted digitalisation of PAE machines for PwS which are subsequently more likely to be acceptable to the user population. The use of NGT enables the capture a range of perspectives which can enrich research design and implementation.

## Conclusion

This article has summarised the application of NGT to facilitate user involvement in the priorities identified at the outset of a programme of research focussed on technological advancement of PAE for PWS. The findings indicated a readiness amongst the stroke rehabilitation community to embrace digitalisation and progress the development of intelligent technologies. Congruence and divergence in the opinions and emphases shared amongst the selected attendees were identified, and perspectives were aligned with different fields of knowledge and experience represented across the expert groups. The importance of an empowering and positive user experience was emphasised by all attendees. The structured discussion and consensus voting generated ideas which will shape the digitalisation of PAE, influence future research and guide implementation of the new technologies.

The perspectives captured from this initial user involvement exercise determined which machines were selected to be prototyped to test the feasibility of the new technology. The vision for the user interface generated during the NGT discussion represented the first stage of the medical device technology framework which underpinned the overall design and feasibility testing programme. The perspectives and ideas captured shaped the subsequent methods implemented to design and prototype the new technology. The prototyped technology will be tested during stages three and four by representative users in laboratory and field settings to determine feasibility and promote an iterative approach towards ongoing development.

## Data Availability

Raw data generated during the PPI events reported in this manuscript are stored in audio and electronic written format at Sheffield Hallam University. Derived data supporting the findings of this study are available from the corresponding author (RY) on request.

## Abbreviations

MDT: Medical Device Technology
PPI: Patient Public Involvement
PAE: Power assisted exercise
PwS: People with stroke
NGT: Nominal group technique
PU: Professional user
EU: Expert user
